# Cervical facet capsular ligament mechanics: Estimations based on subject‐specific anatomy and kinematics

**DOI:** 10.1002/jsp2.1269

**Published:** 2023-06-29

**Authors:** Maryam Nikpasand, Rebecca E. Abbott, Craig C. Kage, Sagar Singh, Beth A. Winkelstein, Victor H. Barocas, Arin M. Ellingson

**Affiliations:** ^1^ Department of Mechanical Engineering University of Minnesota—Twin Cities Minneapolis Minnesota USA; ^2^ Department of Rehabilitation Medicine University of Minnesota—Twin Cities Minneapolis Minnesota USA; ^3^ Department of Bioengineering University of Pennsylvania Philadelphia Pennsylvania USA; ^4^ Department of Biomedical Engineering University of Minnesota—Twin Cities Minneapolis Minnesota USA

**Keywords:** biomechanics, cervical spine, facet capsular ligament (FCL), finite element model, fluoroscopy, videoradiography

## Abstract

**Background:**

To understand the facet capsular ligament's (FCL) role in cervical spine mechanics, the interactions between the FCL and other spinal components must be examined. One approach is to develop a subject‐specific finite element (FE) model of the lower cervical spine, simulating the motion segments and their components' behaviors under physiological loading conditions. This approach can be particularly attractive when a patient's anatomical and kinematic data are available.

**Methods:**

We developed and demonstrated methodology to create 3D subject‐specific models of the lower cervical spine, with a focus on facet capsular ligament biomechanics. Displacement‐controlled boundary conditions were applied to the vertebrae using kinematics extracted from biplane videoradiography during planar head motions, including axial rotation, lateral bending, and flexion–extension. The FCL geometries were generated by fitting a surface over the estimated ligament–bone attachment regions. The fiber structure and material characteristics of the ligament tissue were extracted from available human cervical FCL data. The method was demonstrated by application to the cervical geometry and kinematics of a healthy 23‐year‐old female subject.

**Results:**

FCL strain within the resulting subject‐specific model were subsequently compared to models with generic: (1) geometry, (2) kinematics, and (3) material properties to assess the effect of model specificity. Asymmetry in both the kinematics and the anatomy led to asymmetry in strain fields, highlighting the importance of patient‐specific models. We also found that the calculated strain field was largely independent of constitutive model and driven by vertebrae morphology and motion, but the stress field showed more constitutive‐equation‐dependence, as would be expected given the highly constrained motion of cervical FCLs.

**Conclusions:**

The current study provides a methodology to create a subject‐specific model of the cervical spine that can be used to investigate various clinical questions by coupling experimental kinematics with multiscale computational models.

## Graphical abstract

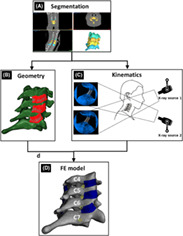

## 1 INTRODUCTION

The cervical spine is a complex structure, comprised of the uppermost seven vertebrae of the spinal column (C1–C7), intervertebral discs, and various other soft tissues. In addition to supporting the head and protecting the spinal cord, the cervical spine is the most mobile spinal region allowing a wide range of head movements and flexibility. Consequently, it can be more susceptible to injury than its thoracic and lumbar regions. Neck pain is a leading cause of activity limitation, affecting 30%–50% of the US population annually.^1^ Sources of neck pain are varied and can be unknown,^2^ but can originate from degenerated joints,^3^, ^4^, ^5^ degenerated disc,^6^, ^7^, ^8^ and/or the ligaments.^4^, ^9^, ^10^, ^11^ The highly innervated cervical facet capsular ligament (FCL), a fibrous connective tissue encapsulating the posterolateral articular facet joints of adjacent vertebrae, has been identified as a potential source of neck pain.^12^, ^13^ Current noninvasive treatments do not offer long‐term relief while invasive treatments have undesirable side effects on the mechanical functionality of the spine.^3^, ^9^, ^14^

Numerous experimental studies have been developed to distinguish the role of the FCL in neck instability and pain.^9^, ^15^, ^16^, ^17^, ^18^, ^19^, ^20^, ^21^ For instance, Quinn and Winkelstein^17^ showed that vertebral retraction simulating exposures similar to those during neck trauma could alter the cervical FCL's collagen fiber organization and was associated with a reduction in ligament stiffness and increase in ligament laxity. Numerous in vitro studies have also described a relationship between excessive elongation of the facet capsular ligament, capsular ligament laxity, and resulting microstructural changes in the capsule fiber organization.^15^, ^21^, ^22^ Whereas experimental data of this sort are an important tool to characterize the FCL's biomechanical and mechanobiological behavior, they do not fully capture the in vivo kinematics experienced by the ligament during normal and abnormal motions in the human. One approach is to develop a finite element (FE) model of the cervical spine, simulating the motion segments and their components' behaviors due to vertebral motions. One objective of FE models is to estimate the stresses and strains in the spinal tissues under various loading conditions since such measurements are difficult to make in experimental examinations.^23^, ^24^, ^25^, ^26^, ^27^ The excessive predicted macroscopic stress and strain within the spinal tissues of such FE models can be used to determine the microscopic tension on the afferent fibers embedded in the FCL^28^, ^29^ and potentially can be associated to pain. Such FE models can ultimately facilitate the development of subject‐specific models that can be adopted as a complementary tool in clinical settings to advance prevention, diagnosis, and treatment plans in cervical injuries. The subject‐specific modeling approach can be particularly attractive when patients' anatomical and kinematic data are available.

Each individual has unique *spinal geometry*, *kinematics*, and *tissue properties*. In order to design a true subject‐specific FE model of the FCL, the specificity of all of those components need to be considered. Recent advances in imaging technologies have led to individualized, image‐based FE models of the cervical spine^23^, ^24^, ^25^, ^27^, ^30^, ^31^, ^32^, ^33^, ^34^, ^35^ capturing the *geometry* effectively. In these studies, the vertebral geometries were reconstructed by processing and segmentation of diagnostic images such as computed tomography (CT) and magnetic resonance imaging scans. The cervical spinal ligaments, including the FCL, however, have been typically represented as two‐node, nonlinear, tension‐only truss or spring elements.^23^, ^24^, ^25^, ^27^, ^30^, ^31^, ^32^, ^36^ Although these simplified representations of the FCL can provide insight into how the ligament affects the motion of the spine, they are not as useful in determining how spinal motion deforms that capsular ligament. Therefore, a higher fidelity 3D volumetric model of the capsule is needed to understand the behavior of cervical FCL during spinal motions.

Realistic boundary conditions on the FCL that accurately reflect individualized in vivo neck *kinematics* are another key aspect in generating a subject‐specific FE model. Despite detailed imaged‐based geometric representation of the cervical spine, many studies have applied a simplified load, typically a rotational moment or a combination of several rotational moments around the vertebra's center of rotation.^23^, ^25^, ^27^, ^30^, ^31^, ^32^, ^34^, ^37^ Although this approach has merit and can provide general insights, it ignores the considerable variation in motion that can occur across individuals.^38^

Unlike geometry and kinematics, it is not feasible to obtain in vivo subject‐specific *material properties* of the cervical FCL. Therefore, FE studies of the spine have relied on soft tissue material properties from the literature. Recognizing the importance of interpatient variability, many studies have investigated the effect of various material nonlinearities^24^, ^39^, ^40^, ^41^, ^42^ on the mechanical response of the FCL and the spine as a whole. These studies used a range of constitutive models, generally treating the tissue as a continuous, homogeneous material. The cervical FCL, however, is a fibrous tissue with complex, heterogeneous collagen fiber organization throughout the tissue, and that organization varies across spinal regions and is recognized to affect the macroscopic‐scale tissue behavior.^39^, ^43^, ^44^, ^45^ Given that subject‐specific properties are not available, it is imperative that the sensitivity of any modeling approach to the choice of constitutive model (homogeneous vs. heterogeneous, single‐scale vs. multiscale) be determined.

Therefore, the goal of this study was to develop, implement, and evaluate a method to create 3D subject‐specific models of the human lower cervical spine (C4–C7), with a focus on FCL biomechanics and to assess the effect of such specificity (geometry, kinematics, and material properties) on the predicted values of strain withing the tissue. This modeling approach incorporates individual spine geometry and replicates individual vertebral kinematics. Because subject‐specific tissue properties were not available, image‐based heterogeneous fiber structures from Quinn and Winkelstein^17^, ^46^ were incorporated in a hybrid multiscale model^47^ to define nonlinear material properties for the FCL. Comparison of the facet capsular strain within the resulting subject‐specific model and generic: (1) geometry, (2) kinematics, and (3) material properties showed that asymmetry in both the kinematics and the anatomy led to asymmetry in strain fields, highlighting the importance of patient‐specific models. We also found that the calculated strain field was largely independent of constitutive model and driven by vertebrae morphology and motion, but the stress field showed more constitutive‐equation‐dependence, as would be expected given the highly constrained motion of cervical FCLs.

## 2 METHODS

### 2.1 Overview

**FIGURE 1:**
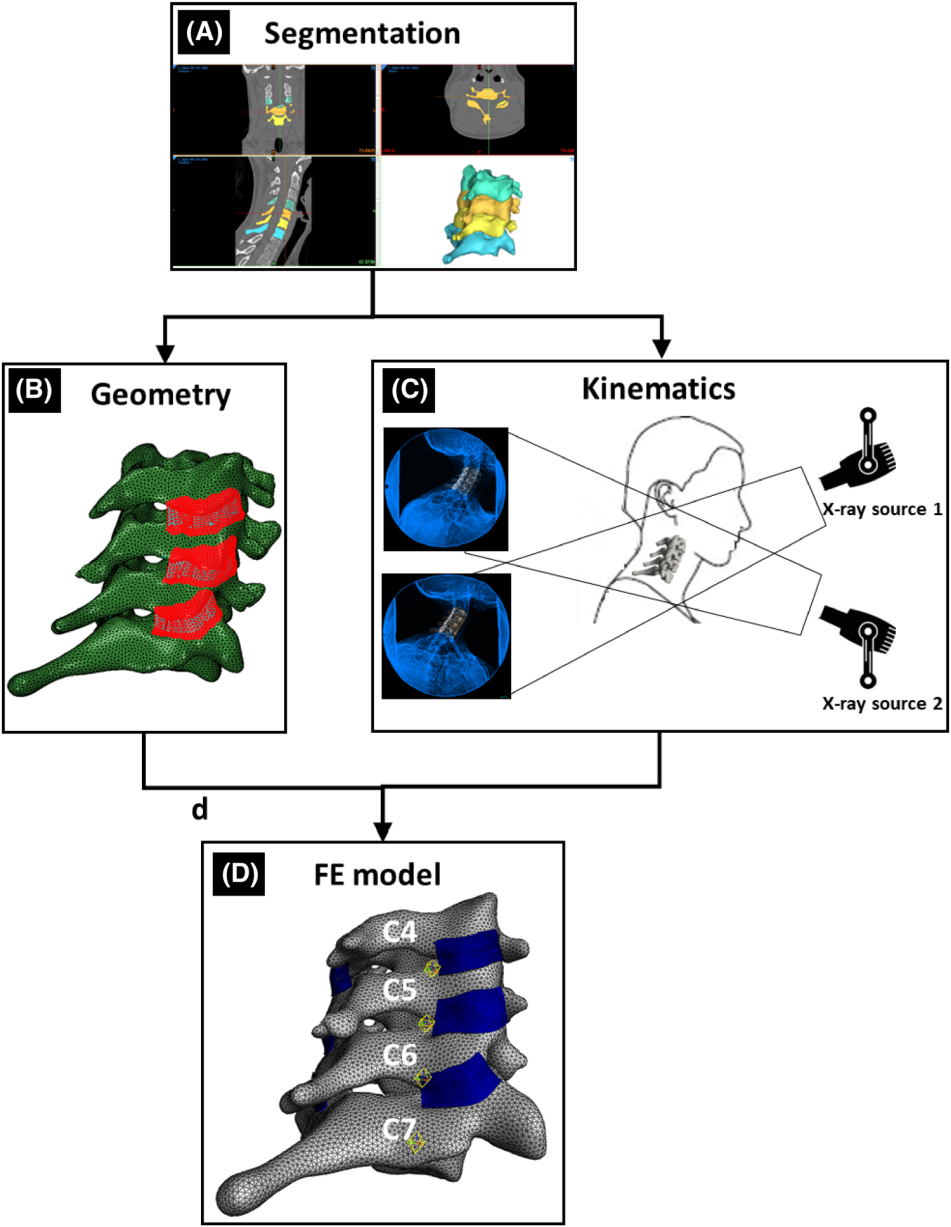
Overview of the methodology to generate a subject‐specific, kinematically driven model of a healthy 23‐year‐old female's cervical vertebrae and facet capsular ligaments (FCL). (A) Bone segmentation to generate the vertebral body. (B) Process of generating 3D geometry of FCLs (for more details, see Figure 2). (C) Biplane videoradiography to extract kinematics for the neck motions. (D) Resulting finite element (FE) model of the lower cervical vertebrae and FCLs.

In this study, a model of a healthy 23‐year‐old female's cervical spine was developed using subject‐specific bony geometry and vertebral motions. The bilateral FCLs were incorporated and executed in FEBio Studio.^48^ Figure 1 briefly illustrates the methodological approach to generate the model. The geometries of vertebrae C4–C7 were created by segmenting the skeletal surfaces from CT scans (0.22 × 0.22 × 0.6 mm; 1.8 mSv; Siemens AG, Munich, Germany) of the participant's cervical spine (Figure 1A). The segmented geometry was then used as an initiation point to generate the FCLs' geometries (Figure 1B), and digitally reconstructed radiographs (DRRs) of C4–C7 were used to extract the vertebral kinematics from biplane videoradiography (Figure 1C).^49^ The fiber structures and material characteristics of the tissue were specified based on available cervical FCL data^17^, ^44^, ^46^ and were incorporated in a hybrid multiscale model^47^ to generate fiber characteristics for the FCLs. The geometry, intervertebral kinematics, and constitutive material models were subsequently imported into FEBio Studio^48^ to form the final FE model (Figure 1D).

The kinematically driven subject‐specific model was compared with a model with neo‐Hookean (NH) material definition, a generic geometry, and a model with mismatched geometry and kinematics (when the geometry of one subject is used with the kinematics of another) to study their respective effect on the model output.

### 2.2 Experimental protocol

The subject‐specific model was created from data collected from a healthy 23‐year‐old female (mass = 49.9 kg and height = 165.1 cm) with no history of neck pain. Consent was obtained in accordance with the University of Minnesota IRB. A cervical spine CT scan was acquired (Siemens AG) with a radiation dose of 1.8 mSv and 0.22 × 0.22 × 0.6 mm voxel size. Kinematic data were collected using a custom biplane videoradiography system (Imaging Systems & Services, Inc., Painesville, OH, USA)^49^ with imaging parameters 160 mA, 70 kV, 30 Hz, 0.16 mSv/trial. The participant performed 3 trials each of axial rotation (AR), lateral bending (LB), and flexion–extension (F‐E) to a metronome set to 50 beats per minute. The participant was instructed to move through her full range of motion for all activities. AR trials began in left AR, moved to right AR, and returned to left AR. LB trials began in left LB, moved to right LB, then returned to left LB. F‐E trials began in full flexion, moved to full extension, and back to full flexion. Summary data of this individual's segmental kinematics are displayed in Table 1.

**TABLE 1:** Segmental range of motion extracted from biplane videoradiography and averaged over three trials of each bending direction for main individual.

	Range of motion (degrees)					
	Left AR	Right AR	Flex.	Ext.	Left LB	Right LB
Head–torso	72.9 (1.3)	−69.3 (1.1)	58.8 (3.1)	−57.7 (1.3)	−39.0 (0.2)	49.9 (0.9)
C4–C5	3.5 (0.5)	−7.8 (0.2)	4.3 (0.7)	−14.9 (0.3)	−7.8 (1.5)	6.2 (0.7)
C5–C6	3.8 (0.9)	−5.4 (0.5)	6.6 (0.3)	−12.7 (1.1)	−8.0 (0.4)	5.4 (0.2)
C6–C7	1.3 (0.2)	−3.2 (0.5)	8.8 (0.2)	−3.5 (2.2)	−4.9 (0.1)	8.4 (0.2)

*Note*: Values are represented as mean (SD).

Abbreviations: AR, axial rotation; Ext. extension; Flex., flexion; LB, lateral bending.

### 2.3 3D volumetric mesh of vertebral bodies

The CT image stack of the participant's cervical spine was imported into image processing software (Materialise Mimics, v23, Materialise NV, Leuven, Belgium), and the 3D vertebral shells of the vertebrae from C4 to C7 were segmented out using an automatic threshold mask based on pixel intensity, followed by manual refinement (Figure 1A). The resulting 3D surface meshes (three‐node triangles—tri3) were created and exported in STL format using a custom MATLAB program. Anatomical landmarks were identified, and local coordinate systems were established for each vertebra. The 3D surface meshes were then re‐meshed using four‐node tetrahedra (tet4) in Materialise 3‐Matic (v15, Materialise NV) to construct volumetric geometries of the cervical vertebrae. The 3D volumetric meshes of vertebral bodies were imported into the FEBio Studio^48^ software as rigid bodies.

### 2.4 Biplane videoradiography and shape‐matching to extract kinematics for the neck motions

Biplane videoradiographic images were undistorted and filtered (DSX Suite, C‐Motion Inc., Germantown, MD, USA), and calibration was performed (XMALab, XROMM).^49^, ^50^ The DRRs of the C4–C7 vertebrae were created from the segmented CT scans. Vertebral kinematics were computed in a semi‐automated shape‐matching software (Autoscoper, XROMM).^51^ Kinematic data were lowpass filtered (fourth order; 3 Hz cutoff), and relative kinematics were extracted.^52^

### 2.5 3D geometries of FCLs

**FIGURE 2:**
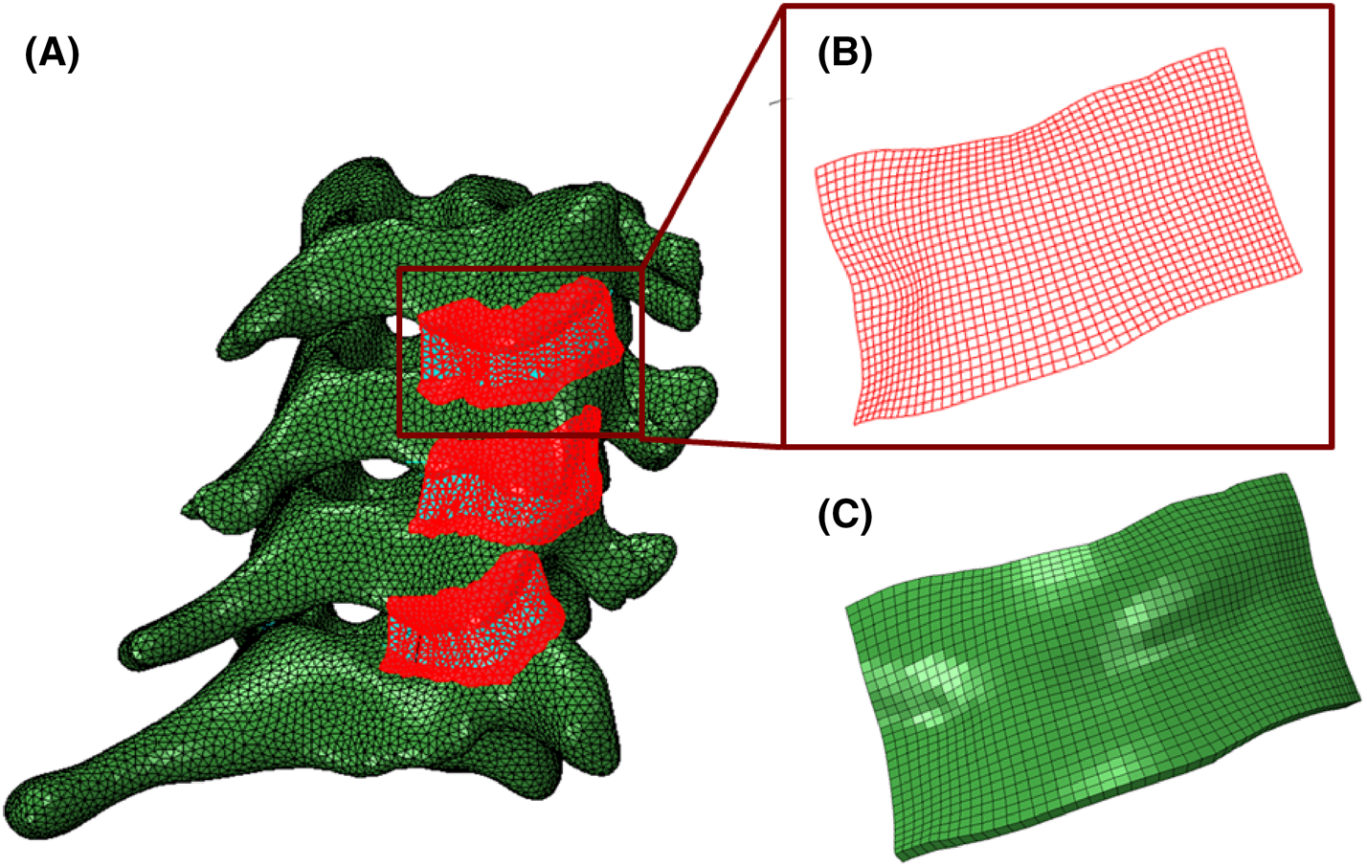
The process of generating 3D geometry of FCLs. (A) Identifying the insertion sites of the FCL into the bone and fitting a surface to encapsulating the facet joints. (B) Structured high‐quality four‐node quadrilateral‐shaped mesh of the right C4–C5 FCL. (C) Extruded eight‐node hexahedron‐shaped mesh of the right C4–C5 FCL.

Extraction of the FCL geometries from participants in vivo is not feasible due to the ligament's small size and low radiopacity. To generate the FCL in this model, we first approximated the insertion sites of the capsule into the bone on each of the inferior and superior facets, as well as the extent of posterior and lateral span (Figure 2A) using CT. This process was performed by two observers (S.S. and B.A.W.) and verified by a third (M.N.); in general, the observers agreed well on the insertion area, and when there was a disagreement, the area with largest coverage was selected, with review and confirmation by both original observers. Next, a surface was generated using Abaqus (R2018, Dassault Systèmes, Vélizy‐Villacoublay, France) to conform to the geometry and to cover the identified regions while encapsulating the facet joint. The method of Liang et al.^53^ was next used to generate high quality four‐node quadrilateral (quad4) elements defining the surface (Figure 2B). That new surface mesh was extruded to an average thickness of 0.42 ± 0.07 mm (mean ± SD)^44^ to generate a volumetric eight‐node hexahedron (hex8) mesh for each FCL (Figure 2C). Finally, the facet capsular geometries were imported into FEBiostudio^48^ such that the insertion areas of the facet joints on each side of the joint conformed to the corresponding areas on the interior side of the FCLs. This was performed with the vertebrae in an unloaded, supine position (CT scan) and served as the reference point for subsequent strain analyses.

### 2.6 Fiber structures and material properties for the FCLs

The cervical FCL is a collagenous tissue with high spatial heterogeneity in its collagen organization.^44^ To incorporate fiber heterogeneity into our continuum FE model, we used a hybrid multiscale method presented elsewhere in detail.^47^ The goal of the hybrid model is to take advantage of the simplicity of continuous FE models in a structure‐based multiscale model to increase efficiency. Details of the simulations to obtain fiber parameters for the constitutive material model are provided Supporting Information Section 1.

### 2.7 Boundary conditions on the ligaments

**FIGURE 3:**
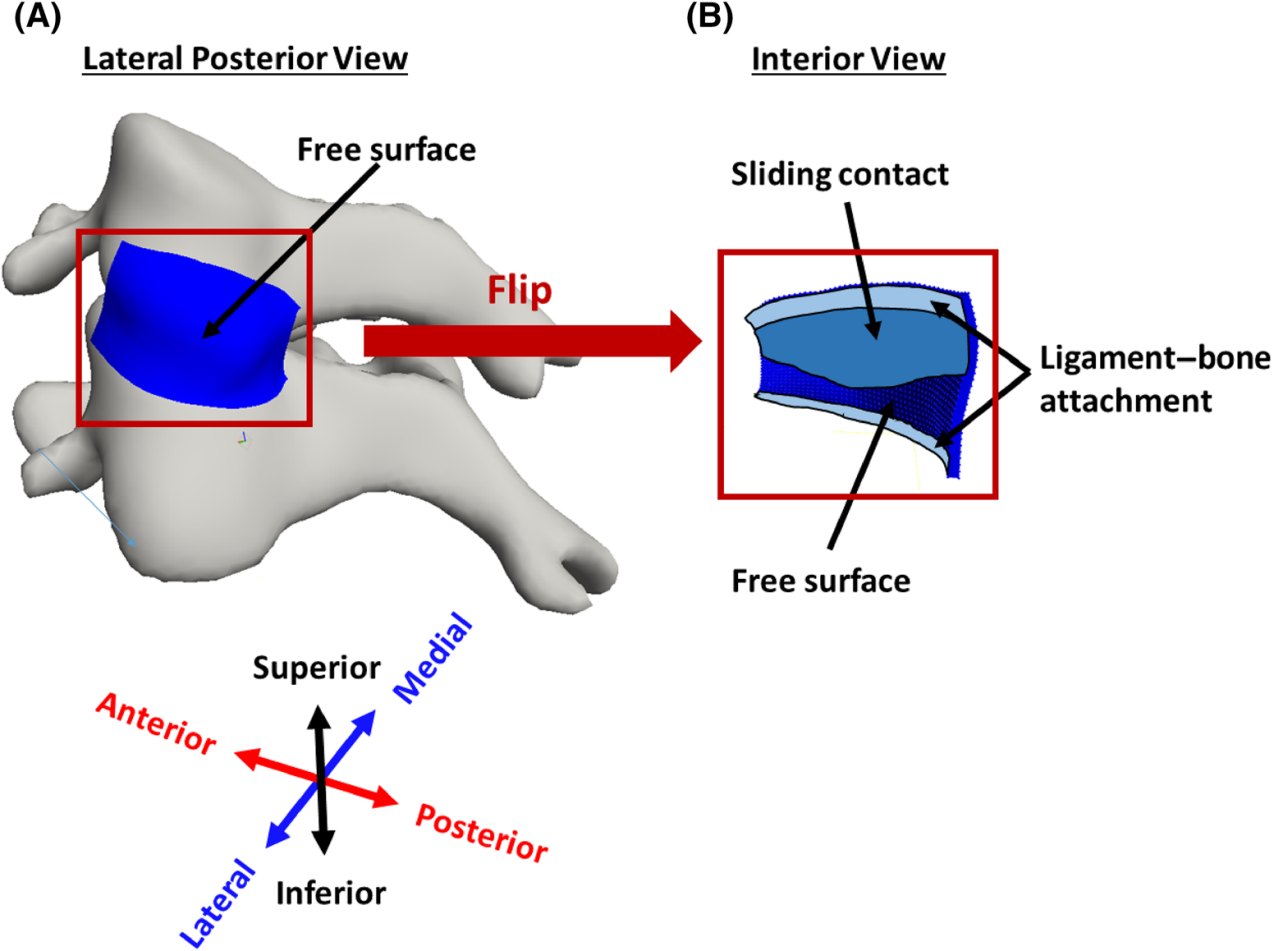
Boundary conditions on the FCL. (A) Lateral posterior view of left C4–C5 FCL, showing that the external surface is entirely free. (B) Interior view of the same ligament, showing rigid attachment to bone (fixed boundary conditions) in the superior and inferior portions of the ligament, sliding contact (free motion parallel to surface but no penetration) where the ligament overlays the bone but is not attached, and free surface where the ligament overlays the joint space.

The portion of the FCL that is in contact with the bony vertebra can be divided into two separate parts: (1) *ligament–bone rigid attachment regions* where the ligament is rigidly attached to the articular pillars of the vertebra and is bound to move with the bone, and (2) a *sliding contact region* where the ligament is in contact with the inferior articular process of the superior facet bone but it is not rigidly bound to the vertebra and can slide over the bone (Figure 3B). Taking this division into account, four different types of boundary conditions were used for the ligament:

- The ligament–bone rigid attachment areas in the interior side of the ligament (Figure 3B) were defined as tied connections controlled by the bone motion. These connections had full kinematic coupling (i.e., displacements *u* ^ligament^ and *u* ^bone^ were forced to be equal).
- Areas of potential ligament–bone contact without attachment were modeled as sliding contact regions (Figure 3B) using the “sliding‐elastic” algorithm in FEBio Studio. The boundary condition allows tangential motion of the ligament along the bone surface but introduces a penalty normal force if the ligament would overlap the vertebral domain.
- Self‐contact was checked for each side of the ligament in case of folding/buckling during spinal motion.

Other surfaces were specified as free surfaces (Figure 3A).

### 2.8 Loading configuration and FE analysis

**FIGURE 4:**
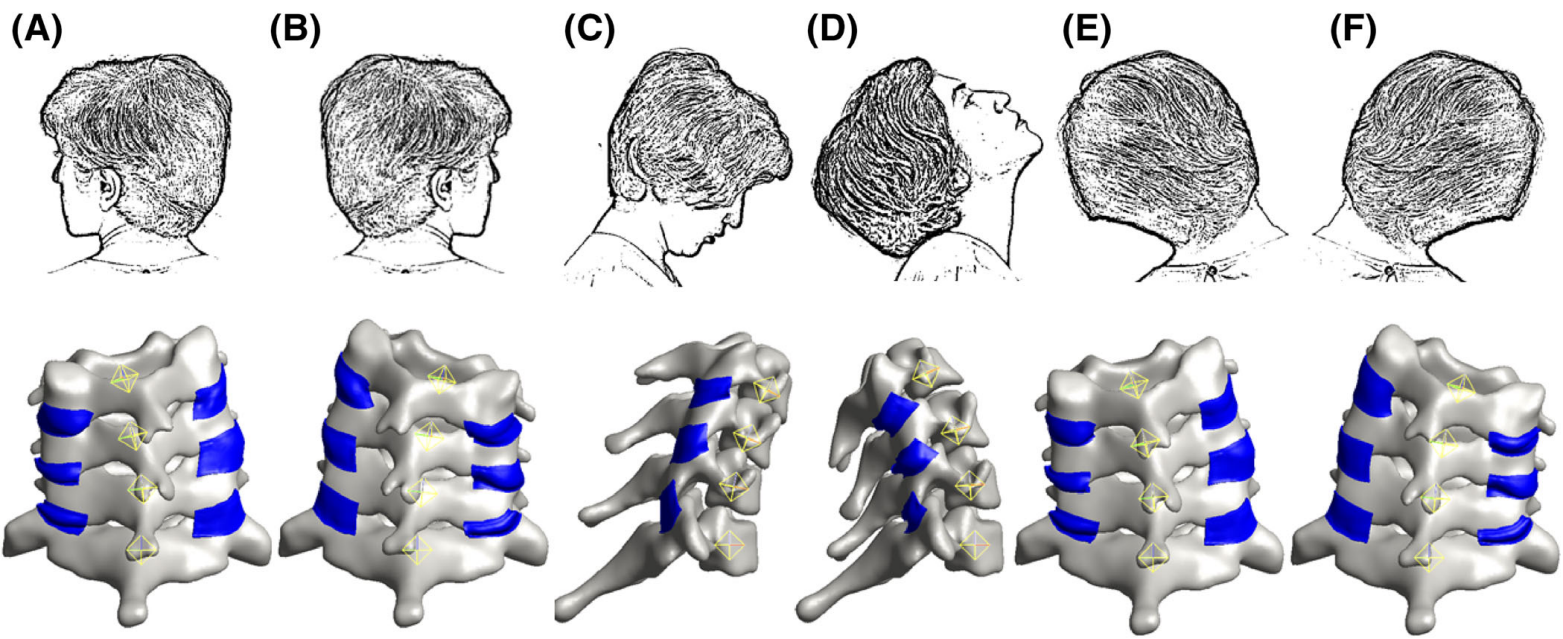
The loading configuration. (A) Axial rotation to the left. (B) Axial rotation to the right. (C) Flexion. (D) Extension. (E) Lateral bending to the left. (F) Lateral bending to the right.

Prescribed displacements were applied to the bone geometries based on the kinematics extracted from biplane videoradiography.^49^ First, the kinematic data were transformed into a fixed‐C7 coordinate system. Then, to simulate each motion in FEBio, the relative motions of C4–C6 vertebrae were calculated. Since FEBio defines the rotation of a rigid body in terms of quaternion angles, the relative transformation matrices measured for each vertebral body were converted to their corresponding quaternion angles and imported into FEBio as load curves. The different loading configurations used in the model can be seen in Figure 4.

Three types of motion were simulated using the FE model: AR to each of the right and left, LB to each of the right and left, and F‐E (three trials each). The first principal strains were recorded for all six FCLs (two on either side of each motion segment) in each trial and were taken to be an acceptable representative measure of model performance. Other metrics of strain may be more relevant to specific questions, but the first principal component offered a reasonable and easily analyzed kinematic output from the model, as well as comparison to prior work in experimental and computational studies. Similarly, first principal stress was taken to be a representative stress metric even though other stresses may, in fact, be more important depending on the question to be answered. The full stress and strain tensors were calculated in the FEBio simulations; we restrict our discussion to the first principal strain and stress for brevity. Based on a mesh convergence study (see below), all computations were performed using hexahedral meshes with an element size of approximately 0.3 mm. Similarly, based on the ligament–bone attachment area sensitivity study below, we chose a model in which four and two rows of elements (a band of ~ 1.2 and 0.6 mm) were attached rigidly to the bone at the superior and inferior ends of each ligament, respectively, for all other simulations.

The model was subjected to a series of sensitivity studies to evaluate its performance and assess the importance of different factors in determining the results. The resulting strain and stress fields were analyzed at the end range of motion in left and right AR and LB and at end range of motion in flexion and extension.

### 2.9 Sensitivity study: Mesh convergence

A mesh convergence study was performed to ensure sufficient refinement of the model mesh. Although this study incorporates bilateral FCLs from C4 to C7, the FCL on the left C4–C5 motion segment was chosen arbitrarily to show the results of the performed mesh convergence study. The coarse mesh had an element size of approximately 0.3 mm (1421 elements). To generate the fine mesh model, each hexahedral element of all ligaments was divided to four elements using FEBio “Refine Mesh” algorithm. Both models were subjected to AR, LB, and F‐E motions, and the first principal stress distribution maps of the simulations were compared to determine the mesh size for the rest of simulations.

### 2.10 Sensitivity study: Ligament–bone attachment area

Since the exact region of ligament–bone attachment has not been defined and is not measurable from the CT scans, we performed a sensitivity study on the effect that the location of this fixed boundary has on the simulated strain within the FCL. For this study, we started with binding the first two rows of elements in the interior surface of the ligament to the superior vertebra (ligament–bone rigid attachment regions) while the rest of the elements were able to slide over the bone (sliding contact region). Then, we enlarged the attachment area in steps of one row of elements until the entire contact area between the ligament and the superior vertebra was represented as a rigid attachment.

### 2.11 Sensitivity study: Material model

The facet capsule ligament is a fibrous connective tissue, comprised of two main regions: (1) a collagen‐rich exterior region and, (2) an elastin‐rich interior region.^54^, ^55^ This unique, heterogeneous fiber structure allows the FCL to undergo large stretches during the neck physiological motions. To investigate the effect of collagen fibers in the output strains and stresses within the FCL, we compared the first principal strains and stresses computed for the FCLs with two different material models: (1) a solid mixture composed of a three‐orthogonal‐fiber‐family material model and a NH ground matrix and (2) a purely NH material. The parameters for the fiber families and NH ground matrix are defined earlier in Section 2.6.

### 2.12 Sensitivity study: Subject‐specific geometry

In the present study, we developed an anatomically detailed, subject‐specific, CT‐based FE model of lower cervical spine. To study the influence of geometric specificity on the output strain patterns within the FCL, we compared the FCL strain results to those obtained using an available generic, geometrically symmetric bone model.^33^ This open‐access model was originally generated based on a 26‐year‐old female subject. After modifying the model to include only the C6–C7 motion segment, the vertebrae were transformed such that C7 vertebra of the generic model aligned with the C7 vertebra of the subject‐specific model (Figure S2a). The FCL geometries were generated as defined earlier in Section 2.5 to match the generic vertebral geometry. The same kinematics as used in the subject‐specific model were applied to the generic model to move the bones. The boundary conditions on the ligaments were as in the subject‐specific model with similar ligament–bone rigid attachment to sliding contact regions ratios.

### 2.13 Sensitivity study: Subject‐specific kinematics

A basic requirement to generate a subject‐specific model is a detailed knowledge of the bone kinematics that drive the model. The boundary conditions for this study were generated based on displacements extracted from a biplane videoradiography of AR, LB, and F‐E of participant's neck. To assess the effect of subject‐specific kinematics, we paired the subject‐specific geometry with the kinematics extracted for a different subject (32‐year‐old female with a mass of 63.9 kg, 172.7 cm height, and no history of neck pain), otherwise following the same computational protocols as above. Summary data of the second individual's segmental kinematics are displayed in Table 2.

**TABLE 2:** Segmental range of motion extracted from biplane videoradiography and averaged over three trials of each bending direction used in subject‐specific kinematics sensitivity study.

	Range of motion (degrees)					
	Left AR	Right AR	Flex.	Ext.	Left LB	Right LB
Head–torso	67.4 (2.0)	−64.3 (5.5)	38.5 (1.0)	−91.2 (2.6)	−48.7 (0.5)	50.6 (1.7)
C4–C5	6.6 (0.3)	−5.7 (0.3)	9.8 (0.3)	−12.1 (0.7)	−8.4 (0.4)	7.49 (0.5)
C5–C6	5.9 (0.4)	−3.6 (1.4)	10.2 (0.4)	−10.2 (0.2)	−2.7 (0.3)	12.2 (0.4)
C6–C7	0.8 (0.2)	−4.4 (1.0)	4.8 (0.5)	−6.4 (0.5)	−11.0 (0.6)	7.1 (0.5)

*Note*: Values are represented as mean (SD).

Abbreviations: AR, axial rotation; Ext. extension; Flex., flexion; LB, lateral bending.

## 3 RESULTS

### 3.1 Mesh convergence study

**FIGURE 5:**
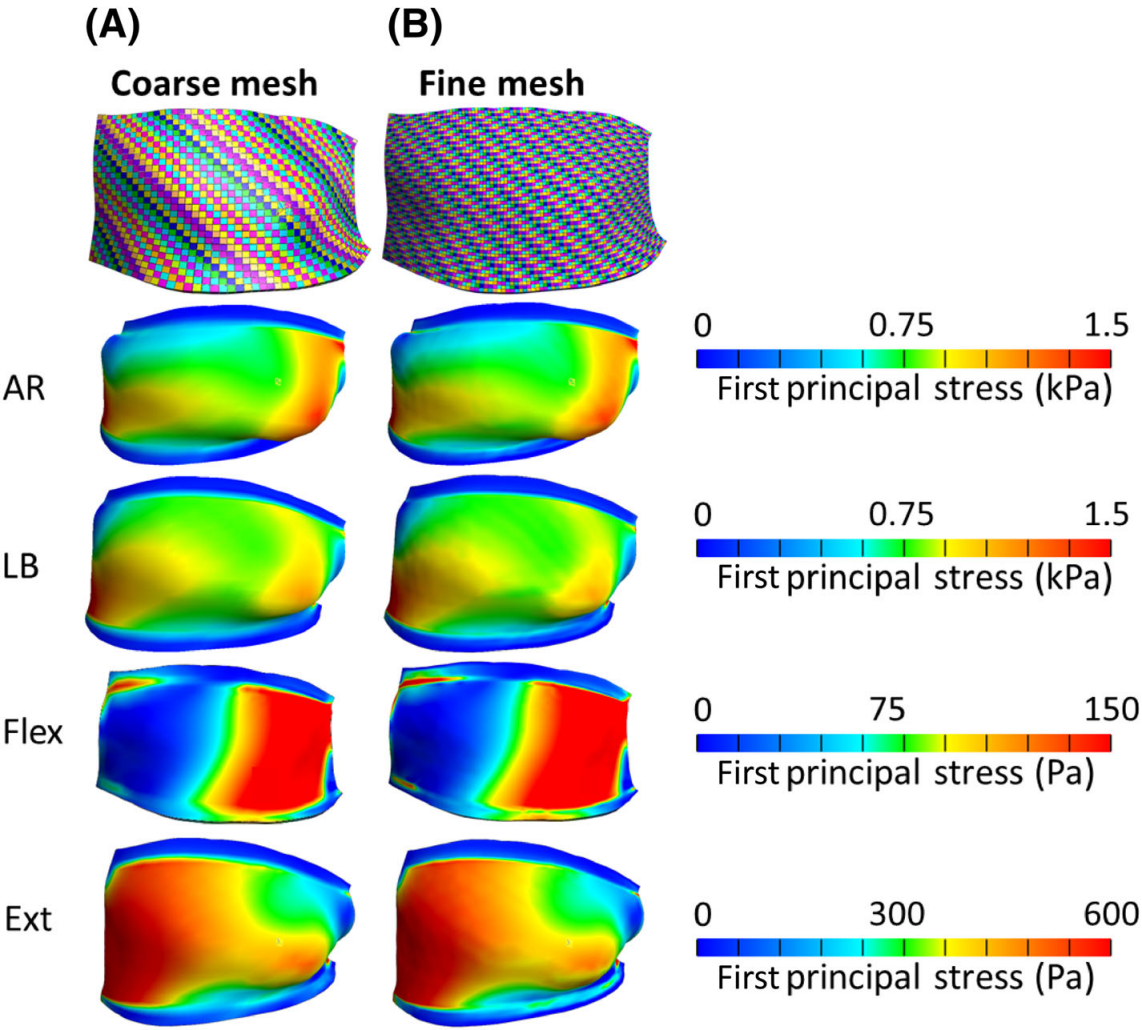
Mesh convergence study on a representative FCL (left C4–C5). The exterior view of mesh visualization and stress distribution maps of the left C4–C5 FCL for (A) coarse mesh and (B) fine mesh models in axial rotation (AR) to left (see part a in Figure 4), lateral bending (LB) to left (see part figure E in Figure 4), flexion (Flex.) (see part figure C in Figure 4), and extension (Ext) (see part figure D in Figure 4) motions.

Although this study considers all the ligaments on both sides of C4–C7 motion segments, all six FCLs in the model experienced comparable strain levels, so it was determined that a single case could be used for mesh convergence studies. The left C4–C5 FCL was chosen arbitrarily as for the detailed analysis. Strain distribution maps of the left C4–C5 FCL for both the coarse and fine mesh model in AR, LB, and F‐E motions are shown in Figure 5. The head is in its end range of motion (AR to left, LB to left, flexion, and extension) in all these representations. The small difference (0.4% in AR, 0.9% in extension, 0.1% in flexion, and 0.3% in LB) of first principal stress between the coarse and fine mesh results in Figure 5 indicates that the refinement of the coarse mesh is sufficient to generate accurate strain maps within the FCL.

### 3.2 Sensitivity study: Ligament–bone attachment area

**FIGURE 6:**
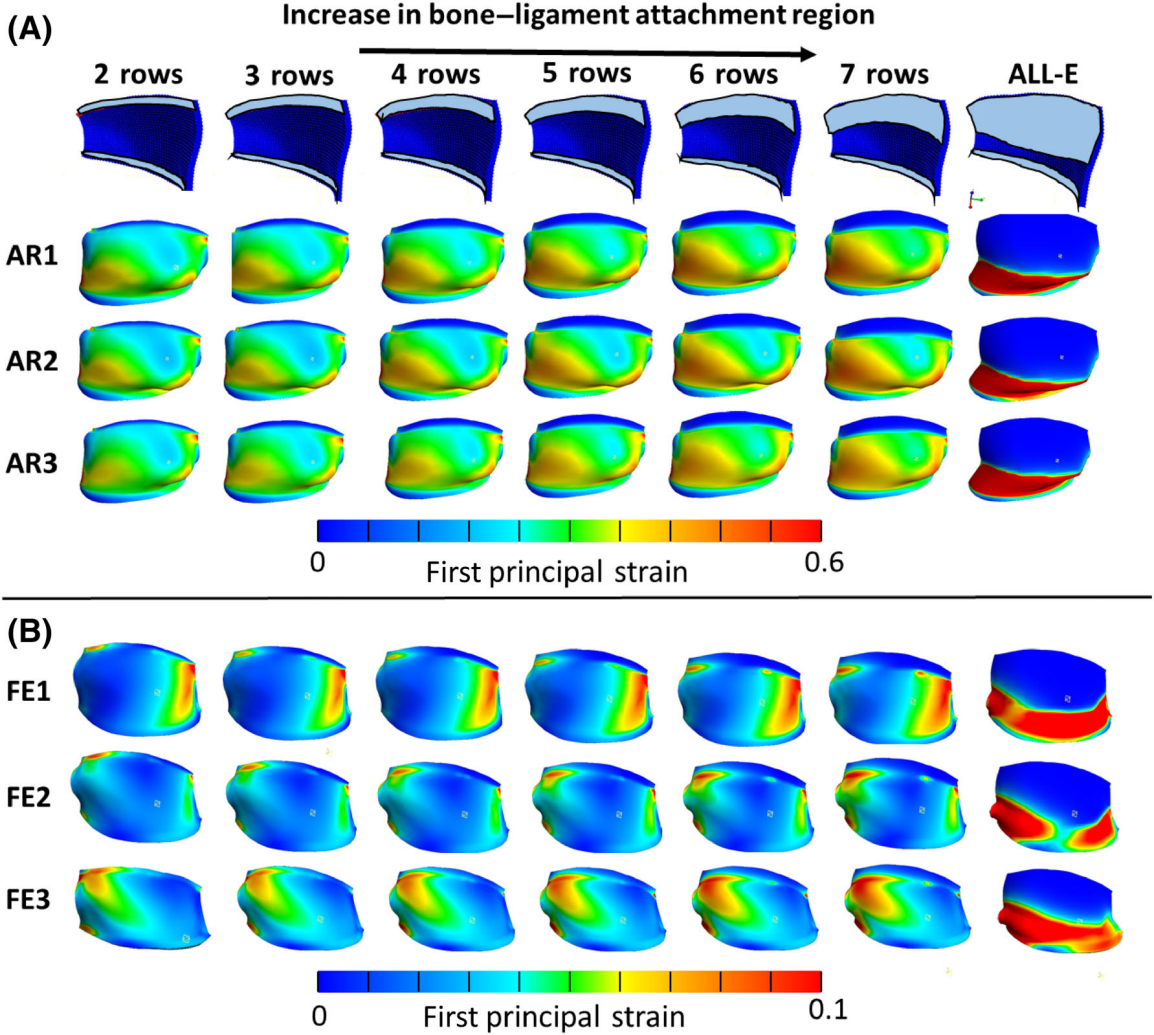
First principal strain distribution maps of the left C4–C5 FCL with different sizes of rigid ligament–bone connection regions. (A) Three trials of axial rotation (AR1–AR3) of head to its end range to left (see part figure A in Figure 4) and (B) flexion–extension (F‐E1–F‐E3) motions when head is in full flexion (see part figure C in Figure 4) (ALL‐E represents a model that the entire contact area between the ligament and the superior vertebra was represented as a rigid attachment).

Out of the six FCLs that were modeled in this study, the left C4–C5 FCL was again selected as a representative model. The first principal strain within the ligament varied with rigid ligament–bone connection area (left to right in Figure 6). Three trials of AR (AR1–AR3) and F‐E (F‐E1–F‐E3) are shown in Figure 6A,B, respectively. The head is in its end range of left AR and flexion, respectively, in all these representations. The first notable feature of the plot is that, despite having identical geometry and constitutive equation for their materials, different trials of the same motion (i.e., different rows in Figure 6) resulted in different strain maps. The difference is most pronounced in the F‐E models, where the kinematics of flexion varied between trials, leading to visible differences among rows F‐E1–F‐E3. In contrast, there was relatively little qualitative change along a given row, indicating that the regions of high and low strain are largely independent of attachment boundary condition, but the strains in the unattached regions increase (shift towards red) with greater attachment area (left to right). In other words, increased contact area leads to increased strain in all unattached regions, with no region clearly increasing more than any other.

**FIGURE 7:**
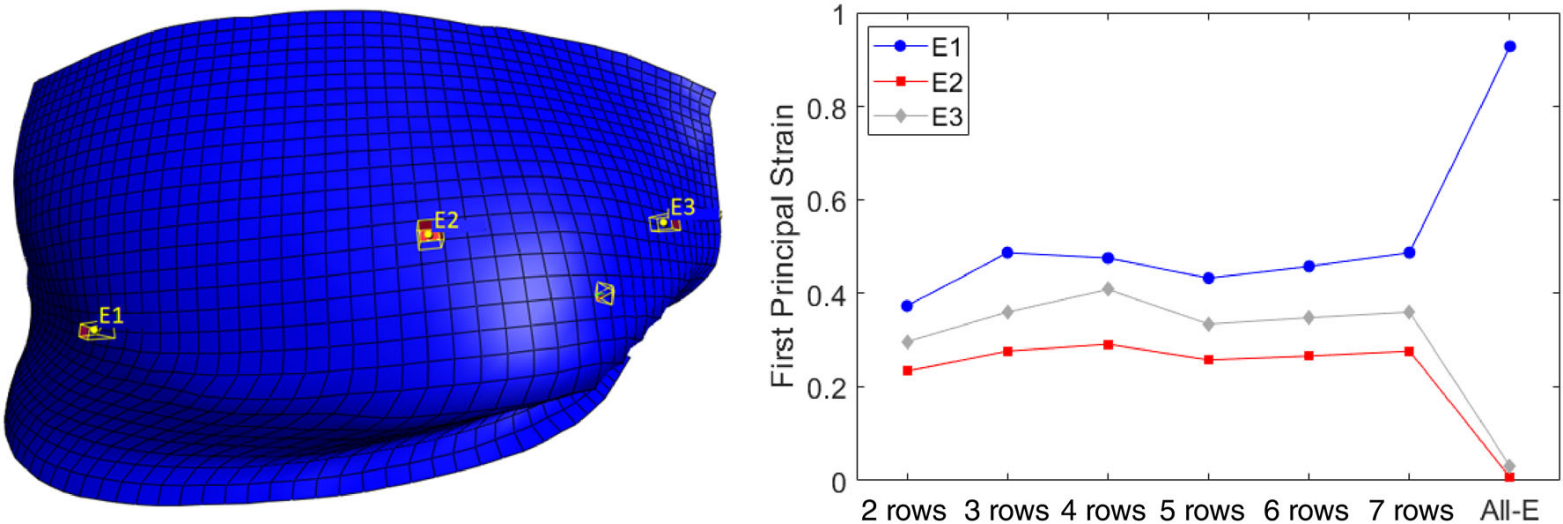
First principal strain vs. number of rows for selected elements (E1–E3) in the left, middle and right side of the left C4–C5 FCL during axial rotation of the neck to its end range to the left (see part figure A in Figure 4).

The increase in the rigid ligament–bone attachment area resulted in slightly higher first principal strain values experienced by the freely moving sections of the ligament (i.e., those that are not attached directly to bone) in all trials of the AR and F‐E experiments. This observation is confirmed by the small upward trend in the first principal strain of the selected elements shown in Figure 7. When the rigid ligament–bone attachment constraint was imposed on all the FCL elements in contact with bone—a nearly certain overestimate of the attachment area—the first principal strain in the free elements increased significantly. The first principal strain in the element marked E1 doubled when the all‐elements‐bonded model was implemented. The drop in the elements marked E2 and E3 in the same model is a result of being rigidly connected to the bone. Figure 7 also shows that the effect of an element's location on the first principal strain is more pronounced compared to the effect of the boundary condition for the more reasonable cases, as the strain increases by at least 60% moving from E1 to E3 compared to 32% increase in E1 strain between the models with the lowest and highest attachment areas.

### 3.3 FE simulations and data analysis

**FIGURE 8:**
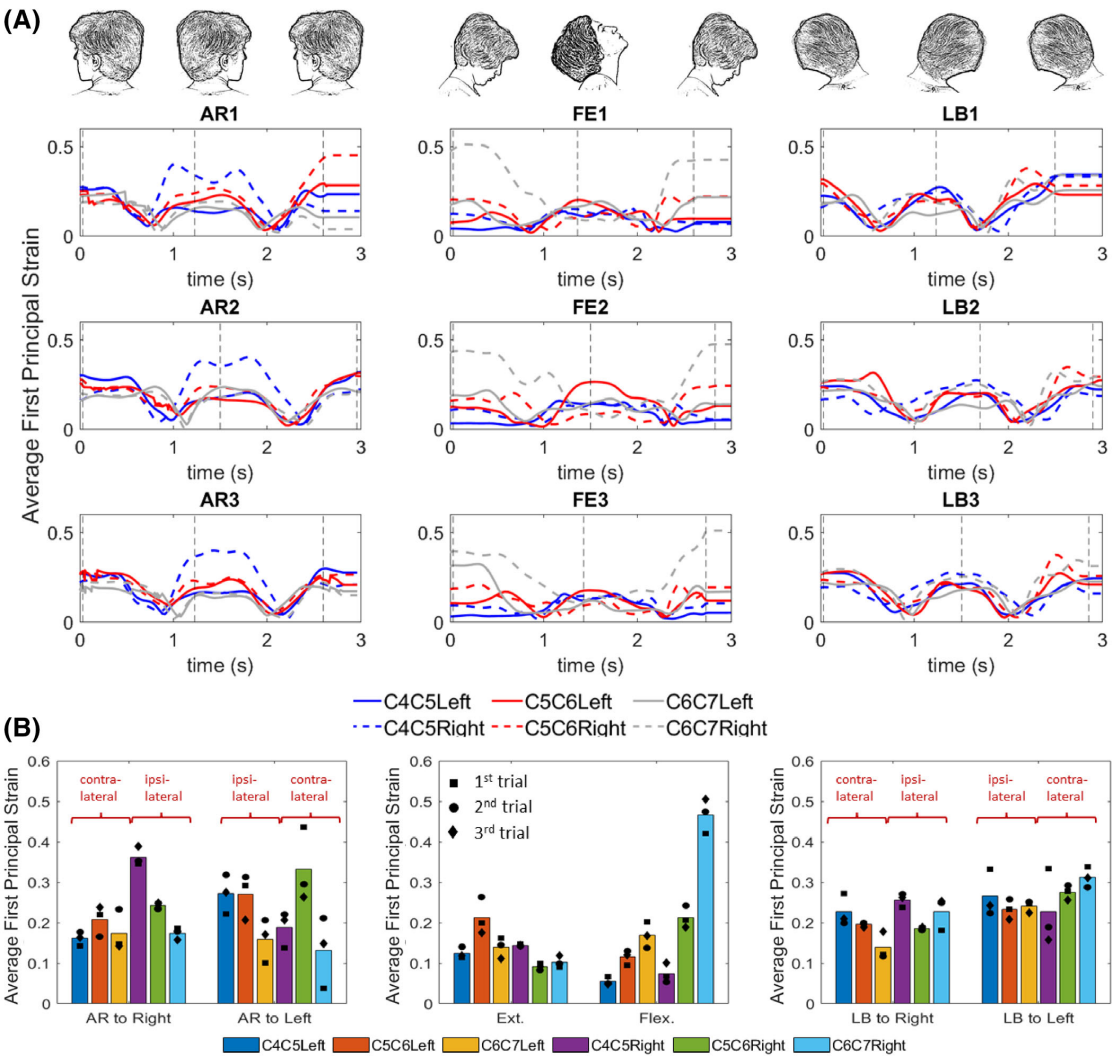
Finite element simulation results. (A) The average of first principal strain within the FCLs of C4–C7 spinal segments during planar motion. Vertical dashed lines in this figure identify when the head was in its end range of motion as is depicted above each column. (B) The pooled average of first principal strain values of (A) over three different trials for each motion type and each facet capsular ligament at the time designated by the vertical dashed lines. The square‐, circle‐, and diamond‐shaped markers in these images represent the exact values for first, second, and third trials, respectively. AR, axial rotation; F‐E, flexion–extension; LB, lateral bending.

As a demonstration of application method, we generated a 3D, subject‐specific FE models of a healthy 23‐year‐old female's cervical spine and simulated AR (AR1–AR3), LB (LB1–LB3), and F‐E (F‐E1–F‐E3) motions. Summary data of this individual's segmental kinematics are displayed in Table 1. The average of first principal strain within the right (dashed lines) and left (solid lines) C4–C7 FCLs is depicted in Figure 8A. The strain values at the end range of motion in Figure 8A were averaged over the three trials to produce an overall average first principal strain for each motion, shown in the bar graphs in Figure 8B. For example, the leftmost dark blue column of the “AR to right” section of Figure 8B is the first principal strain on the left C4–C5 FCL averaged over the three corresponding trials (AR1, AR2, AR3) in Figure 8A at the point of maximum right rotation (marked by a vertical dashed line between 1 and 2 s). The AR and LB to left and flexion motions that are depicted in Figure 8B correspond to the latter incidence of the respective motions in Figure 8A.

As shown in Figure 8A, the right C4–C5 FCL of this individual is more affected by ipsilateral AR toward that facet (panels AR1–AR3), experiencing approximately twice as much strain as the rest of the ligaments experience on average in the same motion. A similar observation can be made for the right C6–C7 FCL in flexion motion (panels F‐E1–F‐E3). As can be seen in Figure 8B, the results were, as expected, roughly symmetric between the right and left sides (e.g., the strain trend within ligaments of right side of C4–C7 motion segments in AR to left is similar to the strain trend within the ligaments of the left side of C4–C7 motion segments in AR to right), but some differences were also observed (the first principal strain was noticeably higher on the right C6–C7 FCL than the left in flexion even though flexion would be expected to produce symmetric FCL stretches).

### 3.4 Sensitivity study: Material model

**FIGURE 9:**
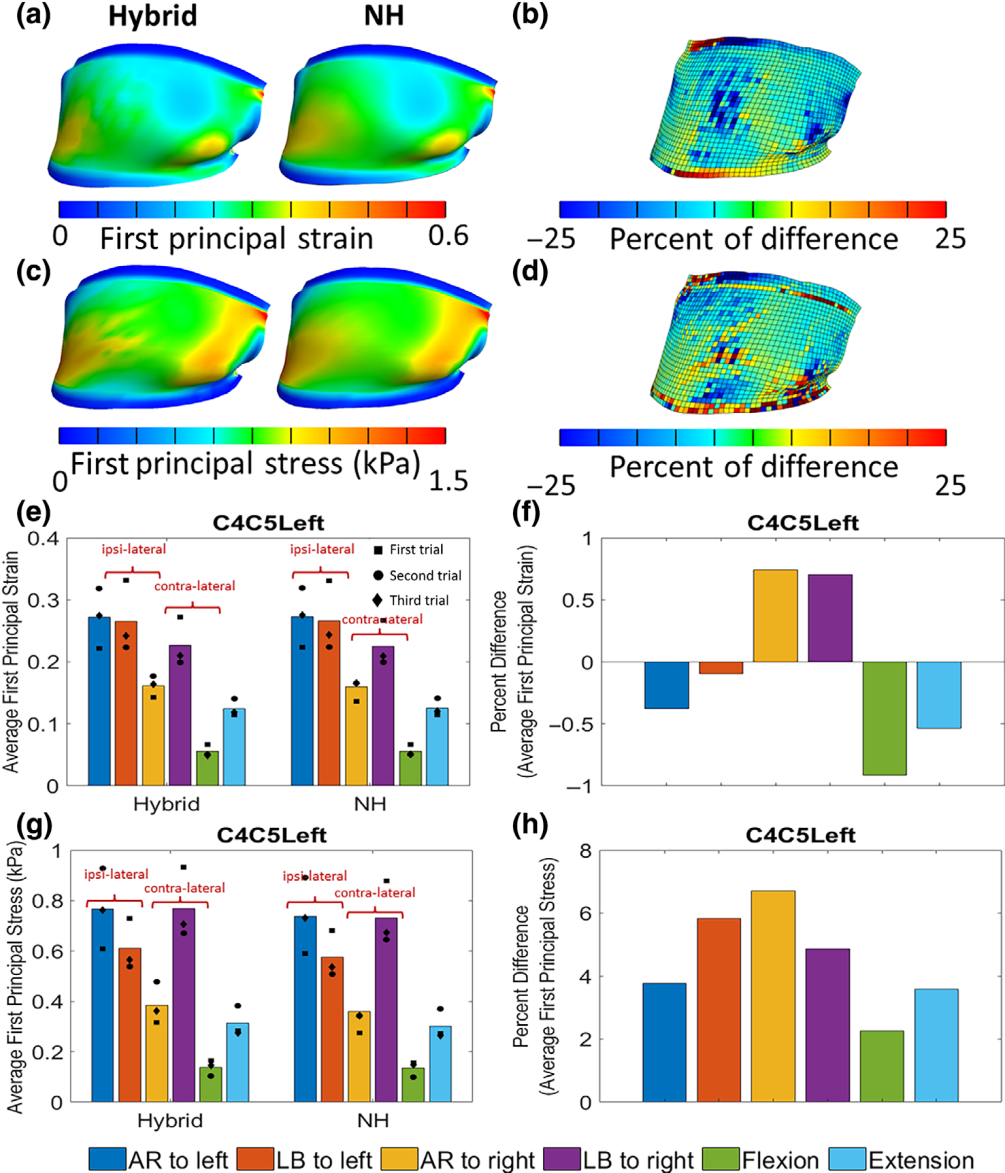
Effect of material on model results. (A) The first principal strain and (C) stress within the left C4–C5 FCL in axial rotation for hybrid and neo‐Hookean (NH) material model when the head is in end range to left (see part figure A in Figure 4). (b) The percent difference of the first principal strain and (D) first principal stress between the hybrid and NH material model shown in (A) and (C), respectively. Average first principal (E) strain and (G) stress within the left C4–C5 FCL in different motions for hybrid and NH material model. The percent of difference of the (F) first principal strain and (H) stress between the hybrid and NH material model shown in (E) and (G), respectively. The square‐, circle‐, and diamond‐shaped markers in these images represent the exact values for first, second, and third trials, respectively. AR, axial rotation; F‐E, flexion–extension; LB, lateral bending.

Although all six FCLs on both sides of C4–C7 motion segments were modeled in this study, for representation purposes, we only focused on the left C4–C5 FCL. Figure 9 illustrates the effects of the material model on the first principal strain and stress distributions within the left C4–C5 FCL for different motions. For the case of AR to left, the first principal strain in the hybrid and NH material models are depicted in Figure 9A with the percent difference between the two (normalized to the strain and stress in the hybrid model) presented in Figure 9B. Similarly, for the AR to left, the first principal stress in the two material models and their percent difference are presented in Figure 9C,D. For all other motion types, the average first principal strain and stress for the two material models and the average percent differences for respective motions are depicted in Figure 9E–H. The strains and stresses in Figure 9E,G are averaged over the entire surface of the C4–C5 FCL first and then over three different trials. The square‐, circle‐, and diamond‐shaped markers in this image represent the exact values for first, second, and third trials, respectively.

The first principal strain and stress distribution maps for hybrid and NH material models (shown in Figure 9A,C) are visually similar. Figure 9B,D shows that 83% of elements defined by NH materials have the first principal strain and stress values within ±10% of those in the respective element defined by hybrid material model. Figure 9E–H demonstrates that the normalized percent difference in the first principal strain resulting from a change in the material model is limited to less than 2% while this difference is more pronounced in the first principal stress by up to 7% as is shown in Figure 9G,H. In comparison with the subject‐specific model with hybrid material definition, the NH material model underestimates the first principal stress and overestimates the first principal strain for all motions except AR and LB to right.

### 3.5 Sensitivity study: Subject‐specific geometry

**FIGURE 10:**
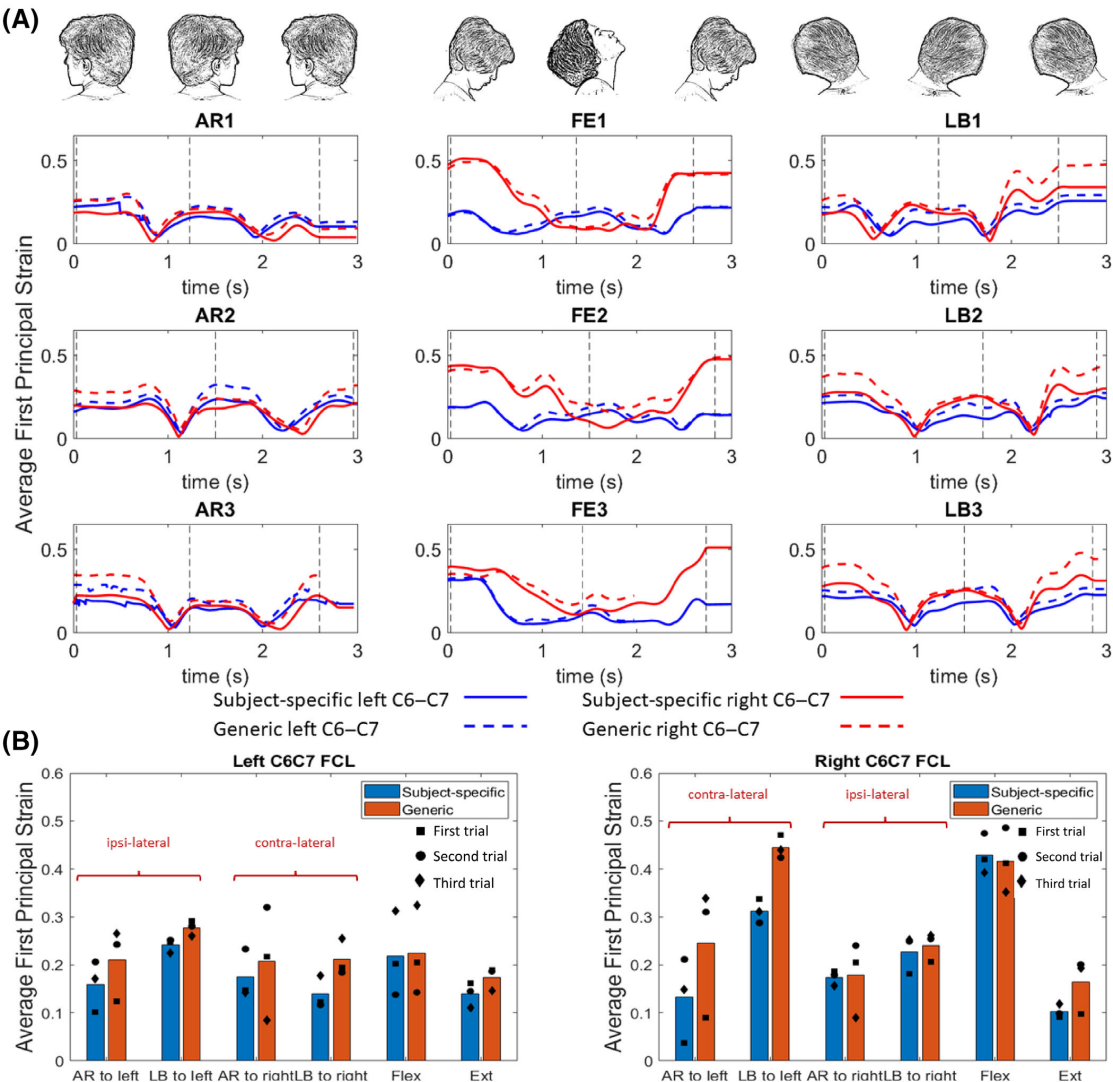
Effect of geometry on model results. (A) The average of first principal strain in a full head motion within the right and left C6–C7 FCL for axial rotation (AR1–AR3), flexion–extension (F‐E1–F‐E3), and lateral bending (LB1–LB3) for a symmetric generic geometry and the subject‐specific model. (B) The average values of ligament‐averaged first principal strains over three different trials for each motion type at the time designated by the vertical dashed lines. Vertical dashed lines in this figure identify when the head was in its end range of motion as is depicted above each column. The square‐, circle‐, and diamond‐shaped markers in this image represent the exact values for first, second, and third trials, respectively. AR, axial rotation, F‐E, flexion–extension, LB, lateral bending.

The effect of geometry on the first principal strain within the right and left C6–C7 FCL is shown in Figure 10. As is shown in Figure 10A, despite having an identical motion pattern as dictated by the kinematics, the generic geometric models consistently overpredicted the average first principal strain. The values in Figure 10B are the average values of ligament‐averaged first principal strains over three different trials for each motion type at the time designated by the vertical dashed lines in the corresponding subfigure in Figure 10A. Due to the mismatch between the generic geometry and subject‐specific kinematics, the generic model failed during the third trial of AR and third trial of F‐E before reaching the end of motion. In the case of AR motion the model failed right after head reached to its end range of motion to left (third vertical dashed line in Figure 10A) so the result of this simulation was used to calculate the averages in Figure 10B. In the case of F‐E motion, the trial was removed when calculating the average value for Figure 10B but was kept in Figure 10A to show the difference between the subject‐specific and generic geometry model.

The difference in average strains is more pronounced in right C6–C7 FCL in AR to left (with 88% difference) and LB to left (with 43% difference) compared to the respective motions to right (4% difference for AR and 8% for LB). The difference between the average strains in left C6–C7 FCL is small to moderate, from 14% difference in LB to left to 52% difference in LB to right motions.

### 3.6 Sensitivity study: Subject‐specific kinematics

**FIGURE 11:**
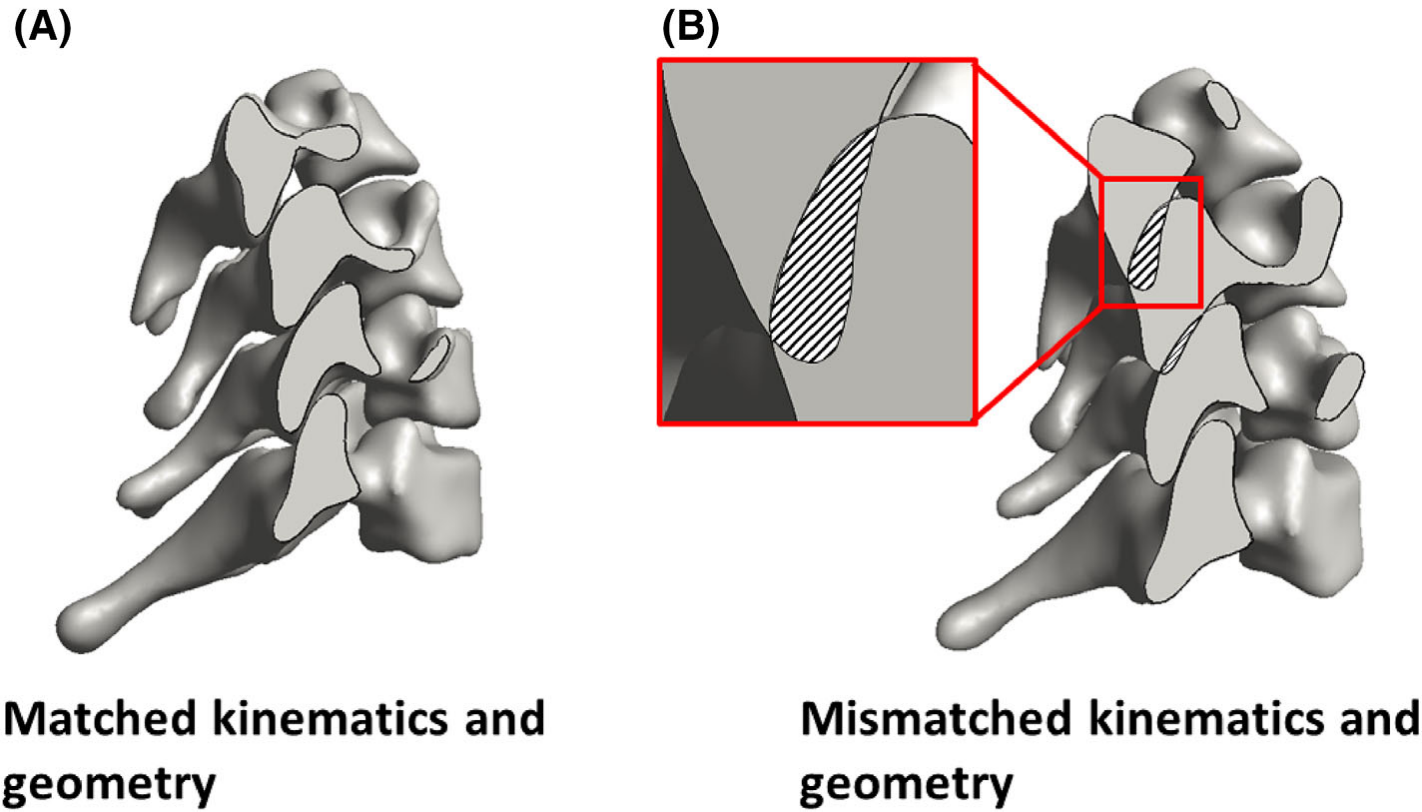
Effect of kinematics on model results. (A) When the geometry and kinematics from the same subject are used, there is no overlap between the vertebrae during extension. (B) When the geometry of one subject is used with the kinematics of another, the inconsistency leads to overlap between the vertebrae during extension. This impossible situation demonstrates the importance of subject‐specific kinematics and anatomy.

Figure 11 illustrates the importance of using the subject‐specific kinematics for the model. As shown in Figure 11A, when the anatomy and kinematics are matched, the vertebrae remain distinct from each other. In contrast (Figure 11B) when a mismatched set of geometry and kinematics is used, the model fails during extension because the inferior articular process/facet of C4 intersects with the superior articular process/facet of C5, a physical impossibility.

## 4 DISCUSSION

In this study, we demonstrated a method to generate a kinematically driven, geometrically accurate, FE model of the FCLs of the lower cervical spine. The model takes as input realistic vertebral anatomy (from CT) and kinematics (from biplane videoradiography). Although the methodology employed herein was applied only to C4–C7, it could be applied to any region of the spine or to other joints if the necessary data and a reasonable estimate of the ligament location were available. The major conclusions from this study, discussed in more detail in subsequent paragraphs, are:

1. the choice of constitutive equation in the model has relatively little effect on the calculated ligament strain field but has a stronger effect on the calculated stress field;
2. although the attachment area between the ligament and the bone must be estimated, the sensitivity to that estimate is relatively small over a wide range of possible areas;
3. integration of both subject‐specific vertebral anatomy and kinematics are critical for estimating the strain in the FCL; and
4. there is notable asymmetry in the strains between the left and right sides, at least for the individual studied.

Two types of constitutive material models (an isotropic NH model and a heterogeneous hybrid microstructural‐continuum multiscale model) were used to estimate the biomechanics of the FCLs during different physiological motions. The NH model was able to accurately estimate the average of the strain and stress values over the tissue (Figure 9E–H). Lu et al.^18^ also showed that the behavior of a goat cervical FCL, loaded under a certain range of a quasi‐static loading condition, can be explained by a linear elastic material model. However, it was shown that a structure‐based material model is needed to obtain the detailed local information for each element (Figure 9A–D) as was also suggested by others.^28^, ^43^, ^56^ Because subject‐specific structural information is not obtainable in vivo, this result points to a limitation of the proposed approach: the ability to describe detailed, small‐scale behavior of the tissue is limited by our ability to describe its small‐scale material properties. For instance, to study the effect of the macroscopic loading mechanisms on the local microscopic structural deformation surrounding a neuron in the ligament, a detailed structure‐based multiscale model would be needed.^28^, ^29^, ^57^ If, however, the overall average strain in the ligament in different physiological motion is one's objective, then a simple NH model could generate an acceptable estimate of strain values. Our results are in agreement with previous studies^43^, ^57^ where they showed that the average magnitude of the axonal loading within a lumbar FCL changes concomitant with the magnitude of macroscopic tissue strain. Therefore, a single‐scale macroscopic model can be used to approximate the magnitude of the axonal loading; whereas, since the location of axonal high‐load regions within the FCL does not collocate with the tissue high‐strain locations, a detailed multiscale, structural model of FCL is needed to determine the location of axonal high‐load regions and would not, with current technology, be available on a subject‐specific basis. Furthermore, the limitations in mapping the fiber orientation of the FCL within the elements of the model should be considered. The FCL is a highly heterogeneous tissue that shows no consistency in its collagen orientation probability density function.^44^ Therefore, it is not realistic to pick a sample that represents the general heterogeneity in the cervical FCL. So, to address this issue, we chose to use the dataset with the median value of the average fiber orientation. For that, a tensor‐based averaging method^43^ was used to calculate the mean alignment strength and fiber orientation for each available sample from a set of previously inferred fiber structures from quantitative polarized images.^17^, ^46^

It is unclear exactly where the rigid ligament–bone attachment ends in a native tissue, and the ligament–bone attachment area may change as humans age and the FCL degenerates because bone growth, calcification of the ligament, and osteophyte formation are common during aging and in disease states such as osteoarthritis.^58^, ^59^, ^60^, ^61^ This bone growth may restrict ligament function, segmental mobility, and eventually lead to boney fusion of the facet joints.^14^, ^58^ Our sensitivity study on attachment area showed that by increasing the rigid ligament–bone connection areas, mildly higher first principal strain values are experienced by the freely moving sections of the ligament (i.e., those that are not attached directly to bone). Perhaps one of the most important outcomes of this study was to provide an approach that can be used to help provide insights on how unique anatomy of each individual, in combination with that individual's specific kinematics, dominates the biomechanics of the cervical spine and its FCLs. For instance, the higher strain average during flexion motion on the right C6–C7 FCL in comparison to the FCL on the left side of this motion segment in subject‐specific model (Figure 8B), for this specific individual, can be attributed primarily to kinematic effects rather than geometry. This conclusion is based on the results of two geometrically different models (subject‐specific vs. idealized symmetric geometry) using the same kinematic inputs and despite having a perfectly symmetric geometry, the same effect has been observed in both models (Figure 10B). Notably, these findings also challenge many previously published cadaveric works that either show or assume symmetric facet mechanics and FCL deformations during F‐E^62^, ^63^—highlighting the value of this work.

These data are important because large strains within the FCL may lead to degradation of the capsular ligament and initiate osteophyte formation,^64^ which reduces the joint's relative motion^58^ and restabilizes the joint's mechanics.^24^, ^65^ Furthermore, these findings are supported by previous studies,^23^, ^36^, ^61^ where they studied the impact of geometry^23^, ^36^ and kinematic^61^ on the biomechanics of the cervical and lumbar spine, respectively. The fact that different trials of the same motion resulted in different strain maps over the tissue (Figure 6), emphasizes the effects that subject‐specific kinematic differences have on the biomechanics of the model since the anatomy is obviously unchanged between trials. Finally, as the result of Figure 11 suggests, the subject‐specific geometry and kinematics are intertwined with each other and having one without the other in a model (for instance, the mismatched kinematic and geometry model of Figure 11B) may result in a physically impossible model (where bones penetrate each other).

To summarize, the current study provides a methodology to create a subject‐specific model of the cervical FCLs (C4–C7) and can investigate various clinical questions by coupling experimental kinematics with multiscale computational models. Extension to other locations and ligaments would be possible if appropriate information could be obtained. Finally, we note that the primary purpose of this study was to explore the method and not to perform a scientific study of spinal motion. Only one subject was examined, and while the data from that subject allowed us to probe our modeling scheme's capabilities and to identify the potential importance of subject‐specific effects, one should not attempt to extrapolate from those results to the population at large. Future works utilizing this workflow may include: (1) investigation of individual or group differences, (2) response to treatment, and (3) extrapolation to other tissues within the spinal column (i.e., cartilaginous surface, intervertebral disc, other ligaments, etc.).

## AUTHOR CONTRIBUTIONS

Maryam Nikpasand, Victor H. Barocas, and Arin M. Ellingson conceived the initial idea for this work. Maryam Nikpasand performed the computational research and model development. Rebecca E. Abbott and Craig C. Kage provided the anatomical and kinematic data for the vertebrae, and Sagar Singh and Beth A. Winkelstein identified the location and rough dimensions of the facet capsular ligaments. Rebecca E. Abbott, Craig C. Kage, Sagar Singh, Beth A. Winkelstein, Victor H. Barocas, and Arin M. Ellingson contributed to conception of the work and to analysis and interpretation of the data. All authors prepared and edited the manuscript.

## FUNDING INFORMATION

This work was funded by the NIH grant U01 AT010326, R03 HD09771, K12 HD073945, and T32 AR050938. In addition, this work was supported by the Minnesota Partnership for Biotechnology and Medical Genomics (MHP IF #14.02) and Promotion of Doctoral Studies (PODS)—Level II Scholarship from the Foundation for Physical Therapy.

## CONFLICT OF INTEREST STATEMENT

The authors declare no conflicts of interest.

## Supporting information


**Data S1.** Supporting Information.
