# Supplementary material for: Cervical facet capsular ligament mechanics: Estimations based on subject‐specific anatomy and kinematics
Source: JOR Spine. 2023 Jun 29;6(3):e1269. doi: 10.1002/jsp2.1269 (PMC10540825; doi:10.1002/jsp2.1269)
Supplement: Supplementary file 1 — Data S1. Supporting Information. [file JSP2-6-e1269-s001.docx]

**Supplementary Material**

**Section1: Fiber structures and material properties for the facet capsular ligaments**

The cervical facet capsular ligament is a collagenous tissue with high spatial heterogeneity in its collagen organization [44]. To incorporate fiber heterogeneity into our continuum FE model, we used a hybrid multiscale method presented elsewhere in detail [47] and described briefly here. First, a set of previously inferred fiber structures from quantitative polarized light imaging (Fig. S1a) [17,46] was used to generate fiber alignment maps (Fig. S1b) [43,57]. The inferred high-resolution fiber alignment maps from quantitative polarized light imaging were morphed to the planar 2D mesh map of each facet capsular ligament (Fig. S1a-f). Then, a tensor-based averaging method was used to calculate the mean alignment strength and fiber orientation for each finite element of the 2D planar facet capsular ligament (Fig. S1g). Next, the 2D planar orientation map was morphed back to the original 3D geometry of each facet capsular ligament (Fig. S1h). The 3D structural information (including the mean fiber orientation and alignment strength) for each finite element was used to generate a nondimensional $1\times1\times1$Delaunay network in MATLAB (R2019a, MathWorks, Natick, MA) to serve as a representative volume element (RVE) in computational domain [66]. Each network contains a set of randomly dispersed 3D seed points representing the network nodes. The edges of Delaunay triangular regions represent collagen fibers and can freely rotate around their contact point at each node. Networks were generated with the global collagen density (the ratio of the total volume of the collagen fibers to the overall network volume) of 0.04 and the degree of alignment equal to the corresponding element’s calculated alignment strength. The global collagen volume fraction of each network was adjusted by changing the number of the initial seed points in relation to the network’s side dimensions while holding the fiber diameter constant for all networks (*100 nm*). The degree of alignment for each network was adjusted by stretching the networks in x-direction until it reaches to the element’s alignment strength. The network was then cropped to a cubic form of $1\times1\times1$ and was checked to maintain the 0.04 collagen density. Finally, the primary network alignment was aligned with the calculated fiber orientation for its corresponding element. The network fibers were modeled with 2-node nonlinear springs:

| $F=AS/B\left[ \exp\left( \frac{B\left( \lambda^{2}-1 \right)}{2} \right)-1 \right]$ | (1) |
| --- | --- |

where *F, A,* S, B and $\lambda$ represent the fiber force, fiber stiffness, fiber cross section area, fiber nonlinearity and fiber stretch, respectively. The values for A and B were 324 *MPa* and 10, respectively [39].

The fiber parameters for a three-orthogonal-fiber-family strain energy density function for each RVE network were calculated using the network-to-continuum scheme described in 38. [47][47][47][47][47][47][47][46][2][40][40][40][39] The goal of the hybrid model is to take advantage of the simplicity of continuous finite element models in a structure-based multiscale model to increase efficiency. Briefly, the hybrid model incorporates the structural information deduced from a full multiscale model into a continuum-based finite element model without the need to run a computationally expensive fully connected multiscale model. To extract the structural heterogeneity of the RVE networks in this model, first, the network undergoes a set of deformations such as x-, y- and z-direction uniaxial stretch, simple shear on the xy-, yz-, and xz-faces, and equibiaxial stretch in the xy-, yz- and xz-plane and then, the macroscopic right Cauchy-Green tensor (*C_ij_ = F_jk_F_ik_*) and the volume-averaged Cauchy stress ($\sigma_{ij}$) [67] are calculated. The volume-averaged Cauchy stress is then converted to the second Piola-Kirchhoff (PK2) stress, *S_ij_*, and is integrated with respect to Green strain, *dE_ij_*, to calculate the total strain energy density function of the network:

| $w^{f}=\int_{E_{ij}=0}^{E_{ij}} S_{ij}dE_{ij}$ | (2) |
| --- | --- |

Then, the network structure tensor, *H_ij_* (Eq. 3) is calculated and used in $\left\langle I_{4} \right\rangle= H_{ij}C_{ij}$ to calculate the square of fiber stretch in the average fiber direction.

| $H_{ij} = \frac{1}{L^{(T)}}\sum_{k=network fibers} L^{(k)}a_{i}^{(k)}a_{j}^{(k)}$ | (3) |
| --- | --- |

where *H_ij_, L^(T)^ , a^(k)^* and *L^(k)^* are the network orientation tensor, total length of all the fibers in the network, orientation of the fiber *k* and length of fiber *k*.

Finally, the strain energy density of the fiber network (w*^f^*) for all test deformations are plotted as a function of the square root of $\left\langle I_{4} \right\rangle$ and is fitted to a three-orthogonal-fiber-family strain energy density function of Eq. 4. This strain energy density function is then used as the fiber component of the total strain energy density function, *W*, in a continuum model to account for the structural heterogeneity of the model (Eq. 5).

| $W^{f}= C_{2}/(2C_{3})\sum_{p=1}^{3} h^{p}\left( \exp\left( C_{3}(I_{4}^{p}-1 \right)^{2})-1 \right)$ | (4) |
| --- | --- |
| $W=W^{m}+W^{f}$ | (5) |

*C_2_, C_3_, h^p^*, $I_{4}^{p}$ represent the fiber modulus, fiber nonlinearity, weighting factor for fiber family *p* and the square of fiber stretch in the direction of fiber family *p*. This fiber-based strain energy density function, *W^m^*, was combined with a neo-Hookean ground matrix (*W^m^* in Eq. 5) in FEBio to form a coupled solid mixture material for each element. The Poisson’s ratio and shear modulus for the neo-Hookean part of material model were 0.48 and 1.2 kPa, respectively [39].

**Section2: Generic vs. subject specific vertebral geometries**

To study the influence of geometric specificity on the output strain patterns within the facet capsular ligament, we compared the facet capsular ligament strain results to those obtained using an available generic, geometrically symmetric bone model [33]. This open-access model was originally generated based on a 26-year-old female subject. The model was modified to include only the C6-C7 motion segment. Then the vertebrae were transformed such that C7 vertebra of the generic model aligned with the C7 vertebra of the subject-specific model (Fig. S2).

**
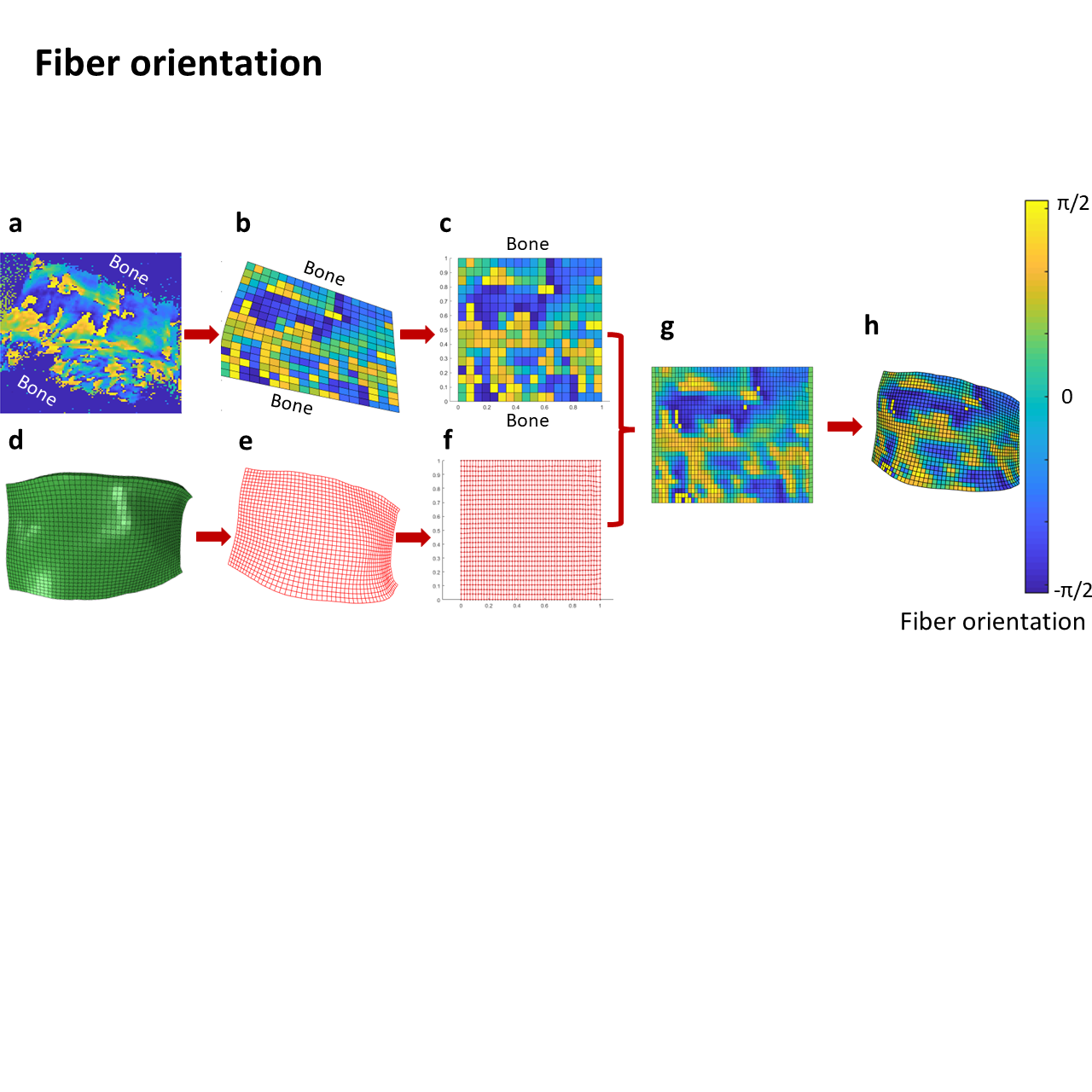
**

**Fig. S1** Steps to map fiber orientation onto facet capsular ligament geometry. **(a)** Inferred fiber structures from quantitative polarized light imaging. **(b)** Fiber alignment maps. **(c)** Mapped 2D planar fiber alignments. **(d)** 3D geometry of the C4-C5 facet capsular ligament. **(e)** Surface mesh of the C4-C5 facet capsular ligament. **(f)** Mapped 2D planar mesh of the C4-C5 facet capsular ligament. **(g)** The fiber orientation map for the 2D planar facet capsular ligament. **(h)** Fiber orientation map mapped to the original 3D geometry of the C4-C5 facet capsular ligament.

**
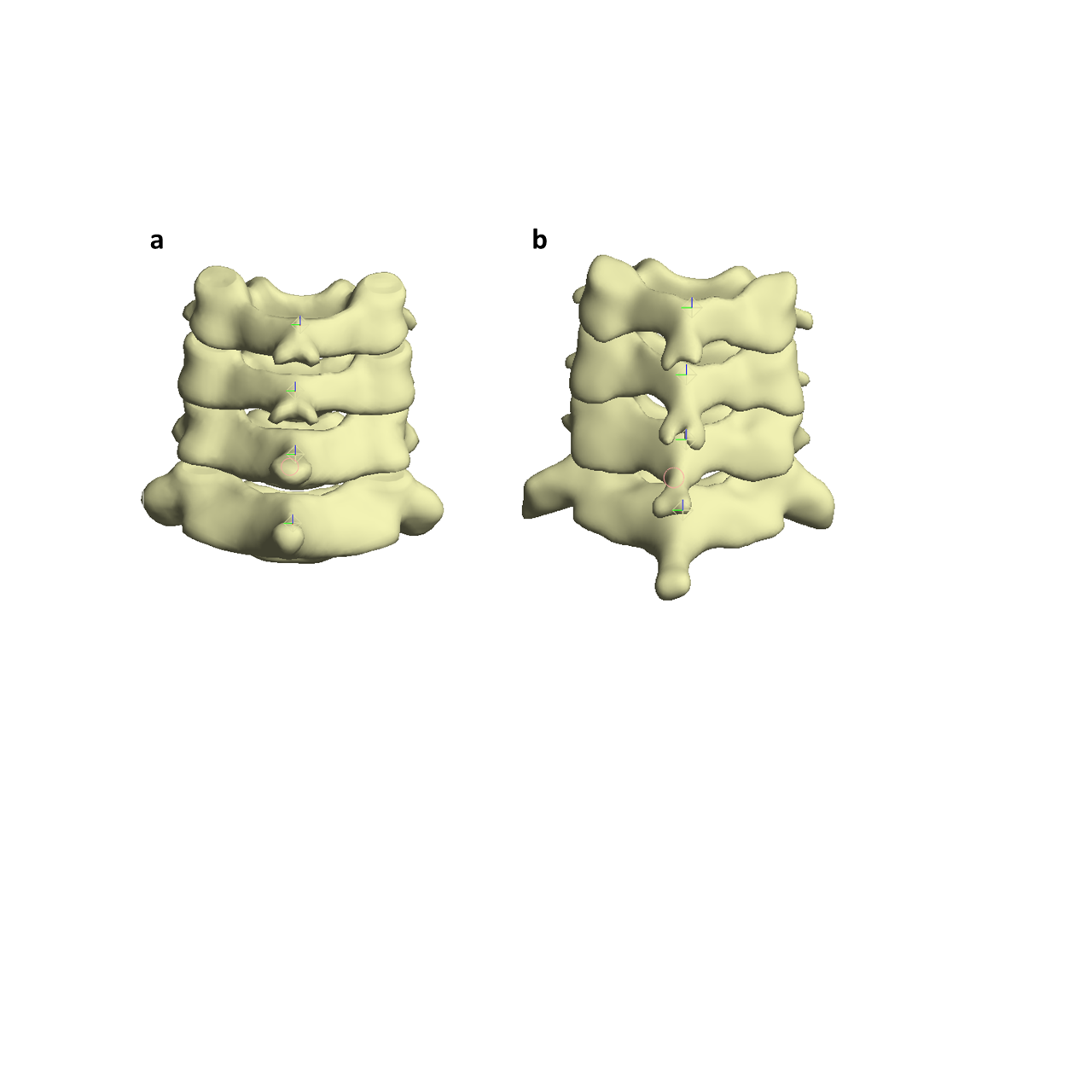
**

**Fig. S2** Vertebral geometry **(a)** Generic geometry model. **(b)** Subject-specific geometry model.
